# Exploring sub-optimal response to tumour necrosis factor inhibitors in axial spondyloarthritis

**DOI:** 10.1093/rap/rkz012

**Published:** 2019-05-04

**Authors:** Fariz Yahya, Karl Gaffney, Raj Sengupta

**Affiliations:** 1 Department of Rheumatology, Royal National Hospital for Rheumatic Diseases, RUH NHS Foundation Trust, Bath, UK; 2 Department of Medicine, University of Malaya, Kuala Lumpur, Malaysia; 3 Department of Rheumatology, Norfolk and Norwich University Hospital NHS Foundation Trust, Norwich; 4 Department of Pharmacology, University of Bath, Bath, UK

**Keywords:** axial spondyloarthritis, TNF inhibitors, sub-optimal response, survival rates, predictors

## Abstract

**Objectives:**

The aim was to define sub-optimal response to TNF inhibitors (TNFi), compare long-term drug survival rates and identify predictors of sub-optimal response in axial spondyloarthritis (axSpA) patients in a UK cohort.

**Methods:**

All axSpA patients attending two centres who commenced TNFi between 2002 and 2016 were included. Routinely recorded patient data were reviewed retrospectively. Patients with paired BASDAI at baseline, 3 and/or 6 months were included for analysis. Sub-optimal response was defined as achieving a ≥ 2-point reduction in BASDAI but not BASDAI50, post-treatment BASDAI remaining at ≥4, and in the opinion of the treating physician these patients demonstrated a meaningful clinical response.

**Results:**

Four hundred and ninety-nine patients were included: 82 (16.4%) patients were classified as having a sub-optimal response; 64 (78%) males, 78 (95.1%) AS and 55/67 (82.1%) HLA-B27 positive. Results are reported as the mean (s.d.). Time to diagnosis was 10 (8.6) years, age at diagnosis was 37 (11.7) years, and age at initiating index TNFi was 48 (11.1) years. Individual index TNFi were Humira (adalimumab, *n* = 41, 50%), Enbrel (etanercept, *n* = 27, 32.9%), Remicade (infliximab, *n* = 5, 6.1%), Simponi (golimumab, *n* = 3, 3.7%) and Cimzia (certolizumab pegol, *n* = 6, 7.3%). The rate of attrition was greater among sub-optimal responders at 2 and 5 years (*P* < 0.05), but not at 10 years (*P* = 0.06), compared with responders. Older age at initiation of TNFi was a predictor of sub-optimal response (odds ratio 1.04, 95% CI 1.01, 1.09, *P* < 0.05).

**Conclusion:**

A significant proportion of patients continued TNFi despite demonstrated sub-optimal response. Further research needs to be undertaken in order to understand this group.

**Key messages**

- A significant proportion of axial spondyloarthritis patients continued index TNF inhibitor treatment despite having sub-optimal response.
- Older age is a predictor of sub-optimal response to TNF inhibitors in axial spondyloarthritis.

## Introduction

The use of TNF inhibitors (TNFi) has been proved to be an effective treatment for most patients with axial spondyloarthritis (axSpA). Patients treated with TNFi have reported efficacy, with significant improvements in pain, disease activity and physical function [1–5]. Serological and imaging parameters also show demonstrable improvements after treatment. Predictors of positive response to TNFi include HLA-B27 positivity, younger age at treatment initiation, widespread inflammation on MRI at baseline and elevated CRP levels [6–8].

Despite the successes of TNFi in treating axSpA, some patients fail to demonstrate a good response to TNFi. The rates of switching first TNFi owing to lack of or loss of efficacy range from 14 to 56% [9]. Older age, negative HLA-B27 and higher baseline BASDAI were reported as predictors of primary inefficacy of TNFi [10]. Although not universally effective for all patients, TNFi use may provide a degree of benefit for patients, which could subsequently influence the decision to continue treatment.

As the use and experience of using TNFi in axSpA increases, it is apparent that a second group of TNFi responders exist. These are patients who respond to TNFi, but the response is sub-optimal. The aim of this study was to define sub-optimal response to TNFi in axSpA, compare long-term drug survival rates and assess predictors in this group of patients.

## Methods

### Patients

A retrospective analysis of axSpA patients (*n* = 499) who commenced TNFi at two specialist centres (The Royal National Hospital for Rheumatic Diseases, Bath and the Norwich and Norfolk University Hospital National Health Service Foundation Trust, UK) between 2002 and 2016 was undertaken, and patients with paired BASDAI [11] at baseline and at 3 and/or 6 months post-treatment were included. TNFi survival data from this cohort have recently been published [12]. All patients had a physician-verified diagnosis of axSpA and were eligible for biologics according to the National Institute for Health and Care Excellence (NICE) criteria (TA383) [13]. There is no formal requirement for clinical databases to apply for ethical review under National Health Service research governance systems, and therefore analysis of anonymized data did not require ethical approval.

Demographic data, including age, sex, date of symptom onset, age at diagnosis, age at TNFi initiation, smoking history, family history of SpA and HLA-B27 status, were recorded. Extra-articular manifestations, including acute anterior uveitis, psoriasis and IBD, baseline BASDAI and individual TNFi drugs, were also recorded. The first initiated TNFi was defined as the index drug. Standard practice in both centres is not to adjust other pharmacological therapies within the first 6 months of initiating TNFi.

### Disease activity and treatment response

BASDAI was assessed at baseline, 3 and/or 6 months after TNFi initiation. AS disease activity score [14] was not routinely calculated, because CRP had not been a prerequisite for TNFi initiation under existing NICE guidelines [13].

We defined sub-optimal response as patients who achieved at least a two (≥ 2)-point reduction at either 3 and/or 6 months from baseline but did not achieve BASDAI50, and BASDAI remained at ≥ 4 at 6 months, and in the opinion of the treating physician these patients demonstrated a meaningful clinical response. However, it is important to note that despite demonstrating a clinical response, the decision to continue TNFi therapy was based on a shared decision between the patient and the physician. The primary outcome was patients achieving sub-optimal response at 3–6 months. All patients who continued on index TNFi treatment after 6 months were identified and included in the survival analysis.

Predictors of sub-optimal response to TNFi were analysed. Patients’ age, sex, time to diagnosis (symptom onset to date of diagnosis), age at diagnosis, age at TNFi initiation, smoking history, family history, HLA-B27 status, presence of extra-articular manifestations, choice of individual TNFi drug, baseline BASDAI and its sub-components were included as variables. Detailed records of concomitant NSAIDs use were not available.

### Statistical analysis

Statistical analysis was undertaken using SPSS Statistics v.22.0 (IBM Corp., Armonk, NY, USA). Predictors of sub-optimal response to treatment were identified. Non-parametric testing, including Mann–Whitney and χ^2^, were used to compare between groups, whereas the Wilcoxon signed-rank test was used for comparison within the group. Survival probabilities were estimated using the Kaplan–Meier method and compared using the log-rank test. Logistic regression analysis was used to assess predictors of sub-optimal response. Multivariable models were used when adjusted for sex, time to diagnosis, age at diagnosis, age at TNFi initiation, smoking history, family history, presence of extra-articular manifestations (acute anterior uveitis, psoriasis and IBD), HLA-B27 status, choice of individual TNFi drug and baseline BASDAI sub-components. Baseline total BASDAI was excluded from the final model because it has a high collinearity with the individual sub-components of BASDAI. A *P*-value of <0.05 was considered statistically significant.

## Results

Four hundred and ninety-nine axSpA patients (AS *n* = 467, 93.6%) who commenced TNFi, and for whom paired BASDAI at baseline and at either 3 and/or 6 months were available, were recruited.

Results are reported as the mean (s.d.). The number of patients who were classified as having sub-optimal response (≥ 2-point reduction without achieving BASDAI50 but BASDAI remained ≥ 4 at 6 months) was 82/499 (16.4%). These sub-optimal responders consisted of 64 males (78%), 78 (95.1%) diagnosed with AS, and 55/67 (82.1%) were HLA-B27 positive. Time to diagnosis was 10 (8.6) years, age at diagnosis was 37 (11.7) years, and age at starting index TNFi was 48 (11.1) years. Extra-articular manifestations included acute anterior uveitis (17/67, 25.4%), psoriasis (14/68, 20.6%) and IBD (7/65, 10.8%). Family history was positive in 12/40 (30%) patients, and 17/70 (24.3%) were active smokers.

**Fig. 1:**
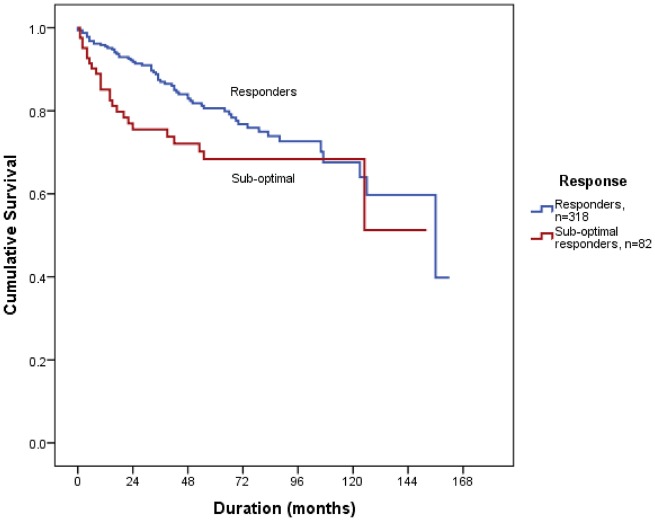
Survival outcome according to response to index TNF inhibitor

The sub-optimal responders were treated for a total of 355 patient-years. Index TNFi used were Humira (adalimumab, *n* = 41, 50%), Enbrel (etanercept, *n* = 27, 32.9%), Remicade (infliximab, *n* = 5, 6.1%), Simponi (golimumab, *n* = 3, 3.7%) and Cimzia (certolizumab pegol, *n* = 6, 7.3%). At 6 months and 1 year, the survival rates among sub-optimal responders who remained on index TNFi treatment were 90.2 and 85.1%, respectively. The rate of attrition was greater among sub-optimal responders at 2 years (75.5 *vs* 91%, log rank *P* < 0.001) and 5 years (68.4 *vs* 79.9%, log rank *P* < 0.05), but at 10 years there was no difference between the sub-optimal responders and responders (68.4 *vs* 67.6%, log rank *P* = 0.064) (Fig. 1). Seventy per cent (58/82) continued index TNFi, despite sub-optimal response, throughout the entire course of follow-up.

Univariate and multivariate analyses of sub-optimal index TNFi response at 6 months are summarized in Table 1. Older age at initiating TNFi was the best predictor of sub-optimal response (*P* < 0.05).

**Table 1:** Univariate and multivariate regression analysis for predictors of index TNFi sub-optimal response at 6 months

Covariates		Univariate analysis		Multivariate analysis	
		OR (95% CI)	*P*-value	OR (95% CI)	*P*-value
Sex, female		1.00 (0.56, 1.77)	0.995	–	–
Diagnosis, AS		1.40 (0.48, 4.11)	0.537	–	–
Time to diagnosis, duration (years)		1.00 (0.97, 1.03)	0.777	–	–
Age at diagnosis (years)		1.04 (1.01, 1.06)	0.001*	1.01 (0.97, 1.04)	0.682
Age at TNFi initiation (years)		1.02 (0.99, 1.04)	0.060	1.04 (1.01, 1.09)	0.023*
HLA-B27, positive		1.19 (0.61, 2.34)	0.607	–	–
Family history, present		1.02 (0.50, 2.05)	0.961	–	–
AAU, present		0.61 (0.35, 1.09)	0.094	0.56 (0.24, 1.30)	0.179
Psoriasis, present		1.14 (0.59, 2.19)	0.689	–	–
IBD, present		0.79 (0.34, 1.86)	0.598	–	–
Smoking	Never	1	–	–	–
	Ex-smoker	1.03 (0.57, 1.84)	0.922	–	–
	Active	0.72 (0.38, 1.38)	0.329	–	–
BASDAI baseline	Total	1.92 (1.56, 2.35)	<0.001*	–	–
	Fatigue	1.39 (1.14, 1.72)	0.001*	1.06 (0.81, 1.38)	0.661
	Spinal pain	1.41 (1.13, 1.75)	0.002*	0.98 (0.70, 1.37)	0.907
	Joint pain	1.29 (1.13, 1.47)	<0.001*	1.18 (0.97, 1.44)	0.086
	Enthesitis	1.33 (1.14, 1.55)	<0.001*	1.04 (0.84, 1.29)	0.726
	Duration^a^	1.49 (1.21, 1.83)	<0.001*	1.15 (0.82, 1.61)	0.414
	Severity^a^	1.14 (1.01, 1.28)	0.026*	1.11 (0.94, 1.30)	0.213
Index TNFi drug	Humira (ADA)	1	–	–	–
	Enbrel (ETN)	1.19 (0.70, 2.03)	0.515	–	–
	Remicade (INFX)	1.04 (0.38, 2.88)	0.931	–	–
	Simponi (GOL)	0.52 (0.15, 1.80)	0.307	–	–
	Cimzia (CZP)	2.33 (0.85, 6.42)	0.101	–	–

AAU: acute anterior uveitis; ADA: adalimumab; CZP: certolizumab pegol; ETN: etanercept; GOL: golimumab; INFX: infliximab; OR: odds ratio; TNFi: TNF inhibitor.

a Related to morning stiffness.

* *P*-value is statistically significant.

## Discussion

This is the first study to define sub-optimal response and describe the characteristics of sub-optimal TNFi responders in axSpA. From a cohort of 499 axSpA patients on TNFi, we have identified 82 (16.4%) patients who achieved a sub-optimal response. Older age at initiation of TNFi predicted this response. Approximately 90% of these sub-optimal responders continued on index TNFi treatment after 6 months.

To our knowledge, there is currently no consensus on the definition of sub-optimal response to TNFi in axSpA. The BASDAI is used widely in clinical practice in the UK to define active disease and as a threshold for eligibility. A cut-off value of 4 has been validated [15–17], and this has been adapted by NICE as a threshold to initiate TNFi treatment [13]. The NICE BASDAI criteria for continuing TNFi have also led to a group of patients being able to continue their treatment despite a sub-optimal response. This is further supported by the published biologic guideline from the British Society of Rheumatology and the British Health Professionals in Rheumatology [18].

In our study, >90% of patients with a sub-optimal response continued on the index TNFi at 6 months. This decision to continue TNFi treatment was at the discretion of the treating physician, which implies that the treating physician considered that they were responding adequately; however, this might have been influenced by switching limitations imposed by NICE guidelines at that time. It is important to note, however, that the option for switching TNFi was available in both centres by local agreement or via an individual funding request process. Studies on continuing TNFi compared with switching in patients with a sub-optimal response are lacking.

We report older age at initiation of TNFi as a predictor for sub-optimal response in axSpA. Younger age has been previously reported as a positive predictor for TNFi response [19, 20], whereas older age was reported as a predictor of non-response [10]. There are a number of reasons why older patients might be more susceptible to a sub-optimal response. It is probable that greater structural changes related to axSpA might be attributable to longer disease duration. In addition, many older patients will have concomitant degenerative spinal disease, the symptoms of which will not respond to TNFi treatment. Finally, the presence of chronic pain syndromes/secondary FM might also contribute to TNFi sub-optimal response in older patients with a longer disease duration [21].

There are limitations to this study. This was a retrospective study based on two centres with limited follow-up data and subjected to selection bias. Missing data might also have contributed to selection bias, because we did not carry out imputation methods during the statistical analyses. The BASDAI collected might not encapsulate the real decision to continue treatment. Data on the presence of peripheral arthritis, enthesitis, CRP, secondary FM, degenerative spinal disease, NSAID use and imaging data were not available for the present study and might have influenced sub-optimal response. One interpretation for continuing treatment in sub-optimal responders is that BASDAI might not be capturing meaningful clinical benefits as determined by patients and physicians. This study also did not include data on all currently available therapies. At the time of the study, IL-17 inhibitors were not available; therefore, there was no option to switch to another mode of action.

In summary, a significant proportion of patients continued TNFi despite demonstrating a sub-optimal response. Older age at starting TNFi is a predictive factor. Further studies are required to understand sub-optimal response to TNFi from the perspective axSpA patients and to investigate the optimal treatment choice for this group of patients.

*Funding*: No specific funding was received from any funding bodies in the public, commercial or not-for-profit sectors to carry out the work described in this manuscript.

*Disclosure statement*: K.G. has received speaker fees, consultancy and/or grants from Abbvie, Celgene, MSD, Novartis, Pfizer and UCB. R.S. has received speaker fees, consultancy and/or grants from Abbvie, Celgene, MSD, Novartis, Pfizer and UCB. F.Y. has received funding for attending congresses, speaker fees, consultancy and/or grants from Pfizer, Janssen, Novartis, UCB and Abbvie.
